# Rapid, Reproducible, Quantifiable NMR Metabolomics: Methanol and Methanol: Chloroform Precipitation for Removal of Macromolecules in Serum and Whole Blood

**DOI:** 10.3390/metabo8040093

**Published:** 2018-12-14

**Authors:** Cora E. McHugh, Thomas L. Flott, Casey R. Schooff, Zyad Smiley, Michael A. Puskarich, Daniel D. Myers, John G. Younger, Alan E. Jones, Kathleen A. Stringer

**Affiliations:** 1 NMR Metabolomics Laboratory, Department of Clinical Pharmacy, College of Pharmacy, University of Michigan, Ann Arbor, MI 48109, USA; mchughce@med.umich.edu (C.E.M.); thflott@med.umich.edu (T.L.F.); crschooff@gmail.com (C.R.S.); zsmiley93@gmail.com (Z.S.); 2 Department of Emergency Medicine, University of Minnesota, Minneapolis, MN 55455, USA; mike-em@umn.edu; 3 Unit for Laboratory Animal Medicine, University of Michigan, Ann Arbor, MI 48109, USA; ddmyers@med.umich.edu; 4 Akadeum Life Sciences, Ann Arbor, MI 48103, USA; john@akadeum.com; 5 Department of Emergency Medicine, University of Mississippi, Jackson, MS 39216, USA; aejones@umc.edu

**Keywords:** pharmacometabolomics, extraction, ultrafiltration, 1d-^1^H NMR, quantitative analysis, preanalytical processing

## Abstract

Background: Though blood is an excellent biofluid for metabolomics, proteins and lipids present in blood can interfere with 1d-^1^H NMR spectra and disrupt quantification of metabolites. Here, we present effective macromolecule removal strategies for serum and whole blood (WB) samples. Methods: A variety of macromolecule removal strategies were compared in both WB and serum, along with tests of ultrafiltration alone and in combination with precipitation methods. Results: In healthy human serum, methanol:chloroform:water extraction with ultrafiltration was compared to methanol precipitation with and without ultrafiltration. Methods were tested in healthy pooled human serum, and in serum from patients with sepsis. Effects of long-term storage at −80 °C were tested to explore the impact of macromolecule removal strategy on serum from different conditions. In WB a variety of extraction strategies were tested in two types of WB (from pigs and baboons) to examine the impact of macromolecule removal strategies on different samples. Conclusions: In healthy human serum methanol precipitation of serum with ultrafiltration was superior, but was similar in recovery and variance to methanol:chloroform:water extraction with ultrafiltration in pooled serum from patients with sepsis. In WB, high quality, quantifiable spectra were obtained with the use of a methanol: chloroform precipitation.

## 1. Introduction

Blood is an ideal biofluid for metabolomics as it is both directly affected by and in contact with organs and tissues and is routinely collected in the clinic [1]. However, if proteins and lipids are not sufficiently removed from the sample before one-dimensional (1-d) proton-nuclear magnetic resonance (^1^H-NMR) spectroscopy, the resulting spectrum cannot be reliably quantified. This is because broad, low intensity peaks produced by macromolecules disrupt the NMR spectrum, obscuring the baseline during spectral processing; reducing the accuracy of quantification for specific metabolites [2]. Although the Carr–Purcell–Meiboom–Gill (CPMG) pulse sequence can be used to reduce the impact of macromolecule peaks on a spectrum, the presence of macromolecules is not simply one of spectral interference [3]. Metabolites may differentially bind to proteins, resulting in signal attenuation which is difficult to quantify. The extent of protein binding can vary between metabolites depending on metabolite and protein abundance as well as the kinetics associated with specific metabolite-protein interactions, further complicating accurate quantification of metabolites in the presence of macromolecules [2]. Thus, for accurate quantitative 1d-^1^H-NMR, it is critically important to both remove proteins from the sample, and to disrupt protein-metabolite interactions before macromolecule removal [2]. 

A variety of methods are used for macromolecule removal from blood samples, including extraction with organic solvents, ultrafiltration, and precipitation with different solvent systems [4,5,6,7,8]. Of these, ultrafiltration and methanol precipitation (MeOH ppt) are widely favored [1]. We have previously reported the use of a methanol:chloroform:water extraction (MeOH:CHCl_3_:water ext) that produces an aqueous fraction for 1d-^1^H-NMR, and an organic fraction for lipidomic analysis by LC-/GC-MS analysis or NMR [4,9,10,11]. However, this protocol is time-consuming and labor intensive, reducing the number of samples that can be assayed at any one time [4]. 

Ultrafiltration (UF) is widely reported as a method of macromolecule removal, and automated profiling programs have even been developed to use with ultrafiltered serum [12], but it has a number of limitations. In addition to the inadvertent introduction of variance by the removal of metabolites bound to proteins, the filters themselves can introduce impurities, primarily glycerol, which necessitates repeated rinses of the filters before use [2,12,13,14,15]. Methanol precipitation offers the advantage of being faster and less resource intensive than MeOH:CHCl_3_:water extractions for similar sample volumes, and has been reported as having superior metabolite recovery [13]. However, studies demonstrating these advantages have tended to rely on pooled serum samples from healthy participants purchased from commercial sources, as well as a relatively small number of samples, which may not capture the overall variability that is likely to occur from a more heterogeneous and larger experimental sample population [4,13,16].

In addition to problems caused by the presence of macromolecules, 1d-^1^H-NMR analysis of the blood metabolome is complicated by the nature of blood as a biofluid. Blood is a metabolically active biofluid which can be influenced by subtle differences in handling before and during macromolecule removal [4,17,18]. Though serum is perhaps the most commonly used blood fluid for metabolomics analysis, it suffers from some problems beginning with its generation, including variability in whole blood clotting time that may influence metabolite concentrations [4,19,20]. Hemoglobin contamination from hemolysis, which can occur in serum and plasma, has been shown to impact the serum metabolome, and is known to be variable, especially in highly heterogeneous and acutely ill populations [4]. These problems can be mitigated by the use of whole blood (WB) for metabolomics. WB provides a more complete picture of the blood metabolome than serum, particularly with respect to energy metabolites, which are not detected in serum. Additionally, because WB requires rapid processing after collection, it is less prone to metabolic changes after collection [4,21,22]. However, there are not yet widely adopted strategies for macromolecule removal in WB. Here, we report a methanol:chloroform precipitation (MeOH:CHCl_3_ ppt) strategy adapted from previously published methods that reduces variance and provides efficient macromolecule removal from WB samples [22].

As accurate quantification of metabolites becomes increasingly important in metabolomics, rapid and reliable pre-analytical sample preparation is necessary [19]. As such, the aim of our work was twofold: first, to further test the efficacy of MeOH ppt under realistic experimental conditions for which we used normal pooled human serum and pooled samples collected from sepsis patients; and second, development of a rapid, highly reproducible strategy for macromolecule removal in WB [4,22]. Here, we rigorously tested methods for the removal of macromolecules from serum and WB to reliably prepare samples for quantitative 1d-^1^H-NMR metabolomics and found notable differences in the efficacy of specific methods in different samples. 

## 2. Results

### 2.1. MeOH Pecipitation with Ultrafiltration Has Better Metabolite Recovery and Lower Variance than Other Methods of Macromolecule Removal in Normal Pooled Human Serum

**Figure 1:**
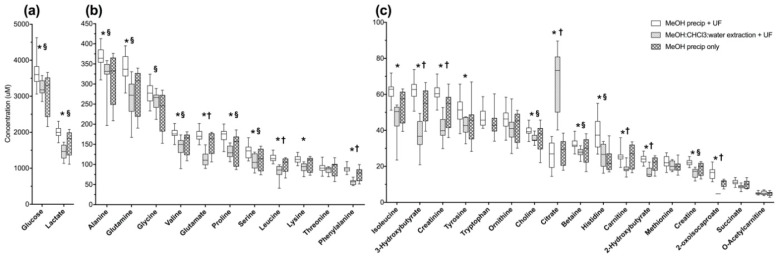
Macromolecule removal method influences measured metabolite concentrations in human serum. Pooled human serum technical replicate samples from healthy individuals subjected to either methanol (MeOH) ppt +UF, or MeOH:CHCl_3_:water ext +UF, or MeOH ppt only, yielded different metabolite concentrations as detected by proton-nuclear magnetic resonance (^1^H-NMR) (500 MHz) spectroscopy (panels **a**–**c**). Box plots represent the interquartile range of all samples in each group (*n* = 20 for MeOH ppt +UF, *n* = 10 for MeOH:CHCl_3_:water ext +UF, *n* = 8 for MeOH ppt samples) with the cross-bar being the median and the whiskers representing minimum and maximum concentrations. (**a**) shows glucose and lactate; (**b**) shows high abundance metabolites with concentrations <500–100 μM; and panel (**c**) shows low-abundance metabolites with concentrations <100 μM. Tukey’s post-hoc test was used to determine significant differences between conditions. * indicates *p* ≤ 0.05 between MeOH ppt +UF versus MeOH:CHCl_3_:water ext +UF; § between MeOH ppt +UF versus MeOH ppt only; † MeOH:CHCl_3_:water ext +UF versus MeOH ppt.

Normal pooled human serum replicates underwent methanol precipitation (MeOH ppt, *n* = 8), methanol precipitation with ultrafiltration (MeOH ppt +UF, *n* = 20), or methanol:chloroform:water extraction with ultrafiltration (MeOH:CHCl_3_:water ext +UF, *n* = 10). A total of 30 compounds were detected and quantified in MeOH ppt and MeOH ppt +UF pooled human serum (Figure 1, Table S1). In MeOH:CHCl_3_:water ext +UF pooled human serum 27 compounds were detected and quantified (Figure 1, Table S1). The compounds that could not be reliably detected in MeOH:CHCl_3_:water ext +UF pooled human serum were 2-hydroxybutyrate, methionine, and tryptophan. A detailed, annotated spectra of a representative MeOH:ppt +UF sample is provided in the supplement (Figure S1). Acetate, ethanol, and methanol were also detected after all strategies, but were excluded from analysis as they are volatile, and all samples were dried by lyophilization. Glycerol was detected in all conditions but was not analyzed as it is a known contaminant from filtration. Isopropanol was also excluded from analysis because it was a contaminant in this lot of pooled normal human serum.

**Figure 2:**
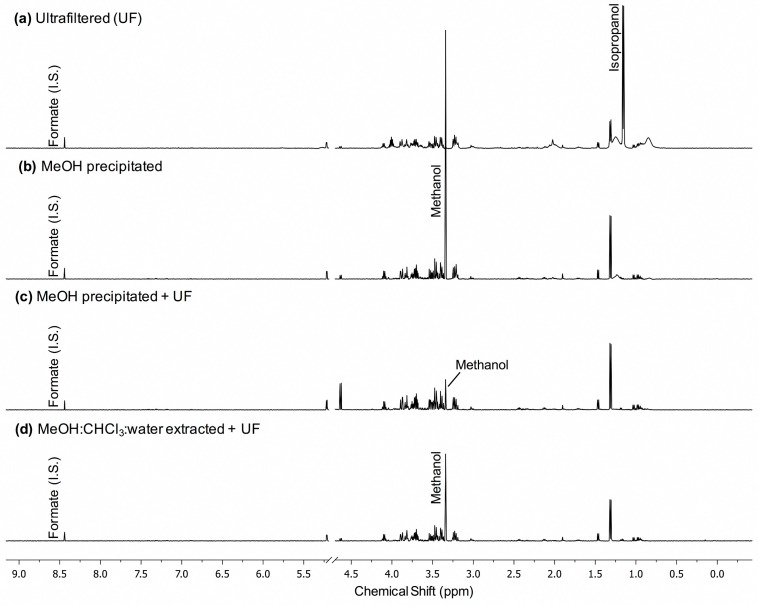
Representative ^1^H-NMR spectra of pooled healthy human serum subjected to a variety of macromolecule removal strategies. Spectra were acquired on a Varian (500 mHz) NMR spectrometer with 32 transients (for pulse sequence, see text). (**a**) Ultrafiltration (UF) alone, (**b**) MeOH ppt alone, (**c**) MeOH ppt +UF, and (**d**) MeOH:CHCl_3_:water ext +UF. Formate was added as the internal standard (I.S.) in all samples. Phase shift correction, excision of the water peak, and baseline correction were performed before identification and quantification of metabolites (see text for details).

Compared to MeOH ppt with and without UF and MeOH:CHCl_3_:water ext +UF, UF alone of human serum did not consistently produce NMR spectra that could be reliably quantified (Figure 2a and Figure S2). This was due to the presence of macromolecules that interfered with the accurate quantification of nearby peaks, which were inconsistently present and varied in intensity between samples (Figure S2). Macromolecule interference was evident in serum that was subjected to MeOH ppt without UF, though at lower intensity than in MeOH:CHCl_3_:water ext samples (Figure 2b). Isopropanol was present at higher concentration in UF samples than in MeOH ppt +UF or MeOH:CHCl_3_:water ext +UF serum because it was partially removed during lyophilization in precipitated and extracted samples, while UF samples were not lyophilized (Figure 2). The presence of isopropanol is likely due to it as an initial contaminant from collection that was not removed during lyophilization (Figure 2a).The NMR spectra of samples after MeOH ppt alone still showed the presence of associated protein peaks, however, MeOH ppt +UF and MeOH:CHCl_3_:water ext +UF produced spectra without evident macromolecule peaks (Figure 2). MeOH ppt +UF of serum did result in more intense peaks than MeOH:CHCl_3_:water ext +UF which is optimal for metabolite identification and quantification (Figure 2c).

Average metabolite recovery was higher in MeOH ppt +UF serum than in MeOH:CHCl_3_:water extractions or MeOH ppt alone, while variance in metabolite concentrations were consistently lower (Figure 1). Total metabolite recovery (TMR, calculated as the sum of the concentrations of all commonly detected metabolites averaged across all samples in each condition) was 17.9% higher in MeOH ppt +UF samples than MeOH:CHCl_3_:water ext +UF (*p* = 0.007), and 14.2% higher than MeOH ppt alone (*p* = 0.075). Additionally, the mean coefficient of variation (CoV ± 95% confidence interval) was lower in MeOH ppt +UF (11.1 ± 1.8%) than in MeOH:CHCl_3_:water ext +UF samples (15.5 ± 1.9%, *p* = 0.012) or in MeOH ppt samples (21.2 ± 1.3%, *p* = < 0.0001). Principal component analysis (PCA) separated the three conditions, and corroborates lower variance in MeOH ppt +UF samples (Figure S3).

ANOVA of normalized concentration data with Tukey’s post-hoc test showed that the concentrations of 23 compounds were significantly different (post hoc *p*-values ≤ 0.05) between MeOH ppt +UF and MeOH:CHCl_3_:water ext +UF; the concentrations of 10 metabolites were significantly different between MeOH ppt and MeOH:CHCl_3_:water ext +UF; and the concentrations of 12 compounds were different between MeOH ppts with and without UF (Table S1, Figure 1). Of these, only the concentration of citrate was significantly higher in MeOH:CHCl_3_:water ext +UF than in the MeOH precipitation conditions (citrate median, IQR: MeOH:CHCl_3_:water ext +UF: 73.3, 25.5 μM; MeOH ppt +UF: 26.9, 11.4 μM; MeOH ppt only: 29.5, 8.1 μM) (Table S1). Citrate concentration was lower in MeOH ppt + UF samples than in MeOH:CHCl_3_:water extracted +UF samples in pooled human serum (*p* = 1.29 × 10^−7^) as well as in human serum from sepsis patients (*p* = 0.086) (Table S2). Citrate is far more soluble in water than in MeOH, suggesting that these differences in recovery could be due to differential solubilities in the two solvents [23]. The concentrations of all compounds that differentiated the two MeOH precipitation conditions were higher in MeOH ppt +UF than in unfiltered samples, likely because of improvements in spectral quality after filtration. 

### 2.2. Differences in Efficacy of MeOH Precipitation with Ultrafiltration in Healthy Pooled Serum and Pooled Serum from Patients with Sepsis are not Due to Effects of Long-Term Storage (−80 °C)

#### 2.2.1. Methanol Precipitation with Ultrafiltration is Not Clearly Superior to Alternate Methods in Pooled Human Serum from Sepsis Patients

**Figure 3:**
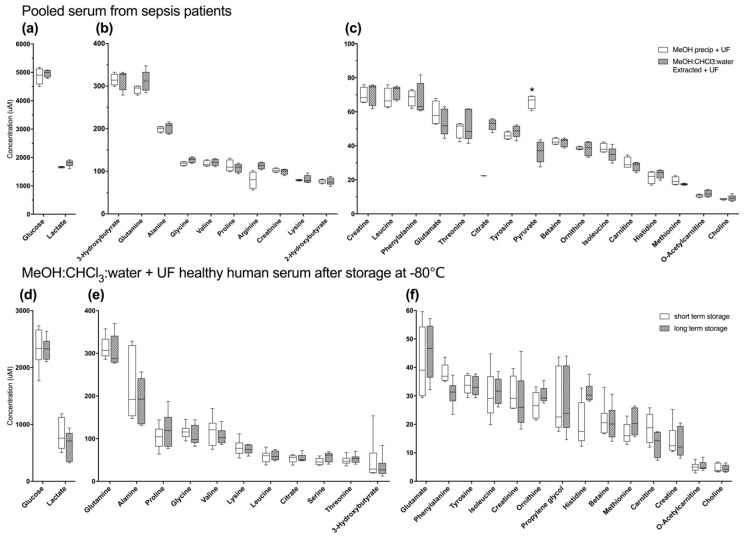
Differences in macromolecule removal strategies were not evident in pooled technical replicates of human serum from patients with sepsis (**a**–**c**) suggesting that sample collection techniques may influence metabolite recovery (*n* = 4 for MeOH ppt +UF, *n* = 5 for MeOH:CHCl_3_:water ext +UF). All samples were also ultra-filtered. Long-term storage at −80 °C did not result in changes to detected metabolomes (**d**–**f**). Box plots represent the interquartile range of all samples in each group (*n* = 6 for both storage conditions) with the cross-bar being the median and the whiskers representing minimum and maximum concentrations. (**a**,**d**) show glucose and lactate; (**b**,**e**) are high abundance metabolites with concentrations <400–100 μM; and (**c**,**f**) are low-abundance metabolites with concentrations <100 μM. * *p* ≤ 0.05 by unpaired Student’s *t*-test of normalized concentration data.

Unlike normal pooled human serum, MeOH ppt +UF was not a clearly superior method for macromolecule removal for pooled human serum that was acquired from sepsis patients. Technical replicates of these samples underwent MeOH ppt +UF (*n* = 4) or MeOH:CHCl_3_:water ext +UF (*n* = 5). A total of 27 compounds were detected and quantified for both conditions. Of these compounds, only the concentration of pyruvate was significantly different (*p* = 0.01), with better recovery using MeOH ppt +UF than MeOH:CHCl_3_:water ext +UF (Figure 3a–c, Table S2). As in pooled human serum, citrate concentration was lower in MeOH ppt +UF human serum from sepsis patients than in MeOH:CHCl_3_:water ext +UF human serum from sepsis patients, though this difference was not significant (*p* = 0.086, Table S2). Representative spectra were similar to those of pooled human serum from healthy subjects (Figure S2). The total metabolite recovery of MeOH ppt +UF (TMR ± 95% confidence interval 8620 ± 558 μM) was not significantly different than that of MeOH:CHCl_3_:water ext +UF (8890 ± 450 μM, *p* = 0.29) unlike the effect seen in pooled healthy human serum. However, there was lower variance with MeOH ppt +UF (CoV: 6.3 ± 1.6%) compared to MeOH:CHCl_3_:water ext +UF (CoV: 9.5 ± 1.7%, *p* = 0.02). PCA confirmed that the metabolomes are distinct, and variance in MeOH ppt +UF is similar to variance after MeOH:CHCl_3_:water ext +UF (Figure S5).

#### 2.2.2. Long-Term Storage at −80 °C does Not Alter the Serum Metabolome

One possible explanation for the different results obtained from normal pooled serum and serum pooled from sepsis patients may be differences in the duration of sample storage prior to macromolecule removal. Samples from sepsis patients were stored 2.5–4.5 years prior to extraction or precipitation, while pooled samples from healthy human controls were stored less than a year before being processed. To test this hypothesis, six matched replicate samples stored (−80 °C) for up to eight months (short-term storage) or for 3–4 years (long-term storage) were subjected to MeOH:CHCl_3_:water ext +UF. 29 Metabolites were detected and quantified in samples after short-term storage, 28 metabolites were quantified in long-term storage samples. Tryptophan was only detected in short-term storage samples (Table S3, Figure 3d–f). Glycerol, acetate, methanol, and ethanol were detected in both conditions but were neither quantified nor analyzed because they are either volatile, or known contaminants from filtration (glycerol). Overall metabolite recovery in long-term storage samples (TMR: 4502 ± 436 μM) was not different than in short-term storage samples (TMR: 4757 ± 816 μM) (*p* = 0.49). Although overall metabolite concentrations trended slightly lower in long-term storage samples, none were significantly different compared to those in short-term storage samples. Also, the average %CoV was not statistically different between short-term (CoV ± 95% confidence interval: 28.41 ± 6.64%) and long-term storage samples (25.39 ± 6.24%, *p* = 0.50). 

### 2.3. MeOH:CHCl_3_:Water Extraction and MeOH:CHCl_3_ Precipitation of Pooled Porcine Whole Blood Yield Similar Metabolite Recovery and Variance

**Figure 4:**
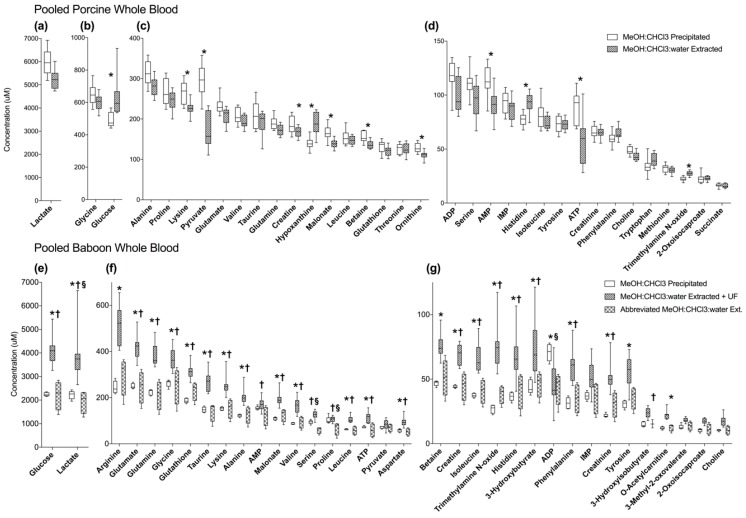
Metabolites (µM) detected by ^1^H-NMR (500 MHz) spectroscopy in pooled pig WB (**a**–**d**) and pooled baboon WB using different macromolecule removal strategies (**e**–**g**). Box plots of samples (*n* = 10 in both pig WB conditions; *n* = 4 for MeOH:CHCl_3_:water ppt and abbreviated MeOH:CHCl_3_:water ext baboon WB, *n* = 10 for MeOH:CHCl_3_:water ext +UF baboon WB) represent the interquartile range with the cross-bar being the median; and whiskers are minimum and maximum concentrations. * indicates *p* ≤ 0.05 between MeOH:CHCl_3_ ppt versus MeOH:CHCl_3_:water ext +UF, § between MeOH:CHCl_3_ ppt versus abbreviated MeOH:CHCl_3_:water ext † abbreviated MeOH:CHCl_3_:water ext versus MeOH:CHCl_3_:water ext.

Due to on-going experiments, we had access to WB samples from two different species, pigs and baboons. In pooled WB from healthy pigs, 36 compounds were detected and quantified after MeOH:CHCl_3_:water ext (*n* = 10); 35 compounds were detected and quantified after MeOH:CHCl_3_ ppt (*n* = 10) (Figure 4, Table S4). Ethylene glycol was detected in MeOH:CHCl_3_:water ext samples, but not in MeOH:CHCl_3_ ppt samples. Ethylene glycol was excluded from analysis because it is a non-endogenous compound and was assumed to be a contaminant. The other 35 compounds were detected in both conditions. Acetone, acetate, ethanol, and methanol were also detected but were excluded from analysis as they are volatile and samples were dried by lyophilization.

The normalized concentrations of metabolites were compared by ANOVA with Tukey’s post-hoc test. Seven metabolites were significantly different (*p* ≤ 0.05) between the two methods. Of these, four had higher recovery in the MeOH:CHCl_3_:water ext condition (hypoxanthine, trimethylamine N-oxide, histidine, and glucose); and three had higher recovery in the MeOH:CHCl_3_ ppt condition (pyruvate, malonate, and lysine) (Figure 4, Table S4). 

Average total metabolite recovery (TMR ± 95% confidence interval) was 9.5% higher in MeOH:CHCl_3_ ppt (TMR = 11400 ± 774 μM) samples than in the MeOH:CHCl_3_:water ext samples (TMR = 10300 ± 580 μM, *p* = 0.02). The quality of NMR spectra produced by both conditions was not noticeably different (Figure S6). In addition, the CoV was not significantly different in MeOH:CHCl_3_ precipitation (11.5 ± 1.1%) than in MeOH:CHCl_3_:water ext (11.7 ± 2.1%) samples (*p* = 0.07). Analysis by PCA also showed that the MeOH:CHCl_3_:water ext condition had very similar variance compared to the MeOH:CHCl_3_ precipitation condition (Figure S7).

### 2.4. MeOH:CHCl_3_ Precipitation Reduces Variance in Baboon Whole Blood Compared to MeOH:CHcl_3_:Water Extractions

Pooled healthy baboon WB was tested to better understand differences in biofluid behavior after undergoing various macromolecule removal strategies. In pooled healthy baboon WB, 41 compounds were detected and quantified after MeOH:CHCl_3_:water ext +UF (*n* = 10), and 39 compounds after blood was MeOH:CHCl_3_ ppt (*n* = 5) (Table S4). An abbreviated MeOH:CHCl_3_:water ext (*n* = 4) was developed, 37 compounds were detected using this strategy. 3-Hydroxyisovalerate, adenosine, methionine, succinate, and threonine were detected in MeOH:CHCl_3_:water ext +UF but not in abbreviated MeOH:CHCl_3_:water ext samples. Adenosine and formate were not detected after MeOH:CHCl_3_ ppt. A total of 35 compounds were consistently detected in all three methods and were compared and used for analysis. Acetone, acetate, ethanol, and methanol were also detected but were excluded from analysis as they are volatile and samples were dried by lyophilization. 

ANOVA with Tukey’s post-hoc test, corrected for multiple comparisons identified differences in the normalized concentrations of 31 metabolites (Table S5). Of these, 27 compounds were significantly different (*p* ≤ 0.05) between MeOH:CHCl_3_:water ext +UF and MeOH:CHCl_3_ precipitations, and 26 compounds were different between MeOH:CHCl_3_:water ext +UF and abbreviated MeOH:CHCl_3_:water extractions. Only lactate, serine, proline, and ADP were significantly different between abbreviated MeOH:CHCl_3_:water ext +UF and MeOH:CHCl_3_ ppt. Only choline, 2-oxoisocaproate, 3-methyl-2-oxovalerate, IMP, and pyruvate concentrations were not different between any of the three conditions. 

Metabolite recovery was highest with MeOH:CHCl_3_:water ext +UF (TMR = 12700 ± 1530 μM), compared to MeOH:CHCl_3_ ppt (*p* = 0.002) and abbreviated MeOH:CHCl_3_:water ext (*p* = 0.002); while recovery was similar in abbreviated MeOH:CHCl_3_:water extraction (TMR = 7049 ± 3040 μM) and MeOH:CHCl_3_ precipitations (TMR=7390 ± 485 μM, *p* = 0.96) (Figure 4e–g). The quality of NMR spectra was similar between the three conditions (Figure S8). 

Though recovery was highest in MeOH:CHCl_3_:water ext +UF samples, PCA shows less overall variance in MeOH:CHCl_3_ ppt than in either MeOH:CHCl_3_:water ext condition (Figure S9). The CoV were calculated for the 35 metabolites common to all three methods and were averaged for each macromolecule removal strategy. Variance (CoV ± 95% confidence interval) was much lower in MeOH:CHCl_3_ ppt samples (8.3 ± 1.3%) than in MeOH:CHCl_3_:water ext +UF (18.1 ± 2.2%, *p* ≤ 0.0001), or abbreviated MeOH:CHCl_3_ ext (29.0 ± 2.4%, *p* ≤ 0.0001). 

## 3. Discussion

High-quality, interpretable 1d-^1^H-NMR spectra are essential for the detection and accurate quantification of metabolites in blood. Here we demonstrate that in human serum, methanol precipitations with ultrafiltration (MeOH ppt +UF) offers effective metabolite recovery, low variance, and efficiency in removing macromolecules. In pooled samples from patients with sepsis we found that MeOH ppt +UF offered similar recovery and variance to MeOH:CHCl_3_:water ext +UF. MeOH ppt +UF is a more efficient protocol, while MeOH:CHCl_3_:water ext +UF offers the advantage of providing a lipid fraction for additional testing. To the best of our knowledge, this study represents the most comprehensive set of experiments assessing the impact of macromolecule removal strategy on quantitative NMR metabolomics. 

Clinical samples, particularly those acquired from highly heterogeneous populations, such as critically ill patients (e.g., sepsis), can introduce unanticipated variance due to differences in sample collection and handling, red blood cell contamination and storage conditions and duration [4]. As such, validation of the reliability of macromolecule removal methods using these types of samples, rather than solely relying on testing using blood from healthy subjects is important and demonstrated by our findings. In pooled healthy human serum MeOH ppt +UF was a superior method of macromolecule removal compared to MeOH:CHCl_3_:water ext +UF. This dramatic difference in metabolite recovery, however, was not replicated in serum pooled from sepsis patients (Figure 3a–c, Table S2). Because blood is a homeostatic biofluid, effects induced by disease states tend to be relatively subtle, so minimizing variance introduced by sample processing is critical for successful analysis and understanding of heterogeneous disease states. MeOH ppt +UF offers both good recovery and consistent measurement of metabolites in serum, particularly when NMR spectra are obtained with a 500 MHz magnet and fewer scans than suggested by some protocols [13,22]. However, MeOH:CHCl_3_:water ext +UF showed similar recovery and variance in pooled samples from patients with sepsis, and results in the production of a lipid fraction, so may be useful for some applications. 

A likely contributing factor to the performance difference found between healthy and sepsis serum was the duration of storage. At the time of assay, sepsis samples had been stored (−80 °C) for 2.5 to 4.5 years (Figure 3a–c). However, further testing did not indicate that differences in the metabolomes of matched samples appeared after long-term storage (−80 °C) (Figure 3d–f, Table S3). This is corroborated by previous investigations that tested the influence of storage (−80 °C) duration on metabolite concentrations [24,25]. Previous studies on the impact of long-term (e.g., years) storage on the metabolome have involved relatively short time frames or have not been performed on replicate samples, instead relying on different samples from similar populations, but have shown that storage affected the concentrations of only a few metabolites [26,27]. Storage time does not appear to contribute to differences in the efficacy of macromolecule removal strategies in pooled serum from normal controls compared to patients with sepsis. However, differences in our results may be due to a smaller n for pooled serum from patients with sepsis (*n* = 4 for MeOH ppt +UF, *n* = 5 for MeOH:CHCl_3_:water ext +UF) than in purchased pooled controls ((*n* = 20 for MeOH ppt +UF, *n* = 10 for MeOH:CHCl_3_:water ext +UF, *n* = 8 for MeOH ppt samples), or simply differences in collection procedures. 

UF is a common technique for macromolecule removal. We found that it was not sufficient to reliably produce quantifiable spectra, as in some cases large protein peaks were present (Figure S2). However, we acknowledge that this conclusion is based on the use of a metnoesy NMR sequence, and a relatively low number of transients (*ns* = 32) acquired on a 500 MHz NMR instrument (Figure 2 and Figure S2). It should be noted that in studies that use UF alone, the use of more scans and employment of higher-powered instruments is common and these strategies are likely to improve spectra quality but may contribute to cost due to longer acquisition times [1,7,12,28,29]. Some groups have also described methods to account for variable performance of filters, both checking for compromised filters, and filtering samples with poor flow-through in a second device, which helps account for inconsistent performance of ultrafiltration alone [12,15]. Nevertheless, it should be noted that use of UF alone could introduce differential metabolite binding to proteins, which may result in variable loss of metabolites depending on metabolite concentration and protein abundance in specific samples [2,13]. 

The CPMG pulse sequence can be used to suppress broad macromolecular resonances. However, this method has limitations because it can cause differential distortion of signal intensities, and most importantly cannot account for differential binding of metabolites to proteins in the sample, which causes signal intensity distortion [2,3,29]. If using the Chenomx library, this is particularly important because it was assembled based on the use of the 1-d metnoesy sequence, and relies on peak shapes that are produced by this sequence. 

The use of WB eliminates many of the pre-analytical processing problems of serum, and captures the red blood cell metabolome, including the energy molecules ATP, ADP, and AMP [4,21]. As WB becomes an increasingly important biofluid for metabolomics, it is important that rapid and reliable methods for its macromolecule removal are developed [4,22]. In both baboon and pig WB, MeOH:CHCl_3_ precipitation resulted in the lowest variance in metabolite concentration. However, the macromolecule removal strategy which resulted in the highest TMR depended on the species of origin of the tested samples (Figure 4). Although no testing was performed on behavior after storage in these samples, it should be noted that pig WB samples were stored in liquid nitrogen after receipt (−196 °C), for less than six months, while baboon WB samples were used after 2–3 years of storage (−80 °C). Furthermore, baboon samples underwent a freeze-thaw cycle during pooling. Freeze-thaw cycles have been shown to impact the serum and plasma metabolomes, so they may have impacted these WB samples [25,30,31]. These different storage conditions might explain the found differences in metabolite recovery, however, we cannot rule out the possibility that differences in sample acquisition procedures contributed (e.g., pig WB was from a commercial vendor). Regardless, speed, efficiency, and the low induced variance due to sample handling make MeOH:CHCl_3_ precipitation an excellent choice for macromolecule removal for WB samples. 

In conclusion, for serum samples, we found that use of MeOH ppt +UF for macromolecule precipitation routinely resulted in high quality NMR spectra for quantitation, while a MeOH:CHCl_3_ ppt of WB produced highly consistent NMR spectra with no evident macromolecule peaks. For high sample volume projects, MeOH:CHCl_3_ precipitation offers better macromolecule removal than MeOH:CHCl_3_:water extraction. MeOH:CHCl_3_ precipitations introduce less variance, are more cost efficient, and allow researchers to complete projects involving large numbers of samples in a shorter amount of time, which is a critical consideration in this metabolically active biofluid. Strategies such as UF can remove macromolecules, but not as completely as precipitation strategies, and metabolites bound to proteins may also be lost [2]. The time-intensive nature of longer macromolecule removal strategies such as MeOH:CHCl_3_:water extractions may significantly limit their use for large metabolomics projects. 

As the field of metabolomics continues to develop, and moves towards greater accuracy in metabolite quantification, it is critical that high quality, reproducible macromolecule removal strategies are developed and tested. This study emphasizes the importance in testing strategies with samples close to those being studied, and that similar biofluids may behave distinctly when exposed to the same procedures.

## 4. Materials and Methods 

### 4.1. Materials and Reagents

Normal pooled human serum from healthy males with AB blood type was purchased from Innovative Research Inc (catalog #IPLA-SERAB-OTC, lot #19421). A second pooled sample was used in later experiments (catalog #IPLA-SERAB-OTC, lot #20636). Pooled normal human serum was stored at −80 °C.

Serum was collected from sepsis patients (*n* = 15) under an IRB approved protocol (Universities of Michigan and Mississippi, HUM00056630) in accordance with the declaration of Helsinki; all subjects gave informed consent for inclusion before participation in the study. All patients had SOFA scores of 8 or higher. Samples were thawed (stored at −80 °C) and 400 μL aliquots from each sample were combined and immediately either extracted or precipitated. 

Serum from healthy human controls (*n* = 6) was collected under an IRB approved protocol (University of Michigan, HUM00038122) in accordance with the declaration of Helsinki; all subjects gave their informed consent for inclusion before participation in the study, samples were stored at −80 °C. 

Baboon WB was collected by venipuncture into glass sodium heparin tubes under an approved animal care and use protocol. Following collection, samples were placed in an ice-water bath. 600 µL aliquots of blood were placed in cryogenic tubes and immediately flash frozen in liquid nitrogen before being stored at −80 °C. For these experiments, 17 samples were thawed and combined (1 mL each) and 550 uL aliquots were generated, then stored before extraction or precipitation (−80 °C).

Pig WB was obtained from Innovative Research Inc (catalog #IR1-070, lot #24307). 600 uL aliquots were generated upon receipt of the blood, and then stored in liquid nitrogen before being extracted or precipitated.

Deuterium oxide (99.8 atom%D), and chloroform (ACS reagent grade), were obtained from Acros Organics (Pittsburgh, PA, USA). Methanol (NF, absolute), monobasic sodium phosphate (monohydrate), and dibasic sodium phosphate (heptahydrate), were obtained from Fisher (Pittsburgh, PA, USA). Calcium formate, deuterium chloride, and sodium deuteroxide were obtained from Sigma Aldrich (St. Louis, MO, USA). DSS-d_6_ (4,4-dimethyl-4-silapentane-1-sulfonic acid) internal standard with 0.2% sodium azide was obtained from Chenomx, Inc. (IS-2) (Edmonton, AB, Canada). 

### 4.2. Ultrafiltration

Pall Nanosep centrifugal devices with a 3kDa molecular weight cut off were obtained from Sigma Aldrich (St. Louis, MO, USA). To remove glycerol and other residues from the manufacturing process each filter was rinsed by adding 0.5 mL ultrapure water followed by centrifugation (14,000× *g*, 4 °C for 4 min). Filters were rinsed five times with ultrapure water and three times with deuterium oxide to minimize water interference in NMR spectra. After each rinse, the filtrate was discarded, between water and deuterium oxide rinses and after the final rinse, any excess water was removed from the top of the filter. 

The total volume of each sample (up to 500 μL) was added to a filter. Filters were centrifuged (14,000× *g*, 4 °C for 20 min) and the filtrate was transferred to a cryovial. To recover remaining metabolites and disrupt any proteins on the surface of the filter, 50 μL of deuterium oxide was added to the top of each filter, filters were vortexed (10 s) and centrifuged (14,000× *g*, 4 °C for 25 min). Additional filtrate was added to cryovials for analysis. 

### 4.3. Methanol Precipitations (MeOH ppt)

This protocol was modified from a previously reported methanol precipitation strategy [13]. Samples were thawed in an ice-water bath. A small volume (20 μL) from each sample was stored (−80 °C) for future assays. 500 μL of each sample was transferred to a microcentrifuge tube. Methanol was added to achieve an approximate 1:2 sample:MeOH ratio. Samples were vortexed, then chilled at −20 °C for 20 min followed by centrifugation (13,400× *g*, 4 °C for 30 min) to pellet macromolecules. Supernatants were decanted to clean microcentrifuge tubes and dried by lyophilization. Dried samples were resuspended in D_2_O (500 μL) to undergo UF as described above or for immediate NMR assay. 

### 4.4. Methanol:Chloroform:Water Extractions (MeOH:CHCl_3_:Water Ext)

Samples were thawed and glucose measured as described above. A dual-phase MeOH:CHCl_3_:water extraction was performed as previously described, with some modifications (detailed procedure provided in supplement) [9,10]. After lyophilization samples were resuspended in 500 μL D_2_O for UF or NMR analysis. 

### 4.5. Abbreviated Methanol:Chloroform:Water Extractions (MeOH:CHCl_3_:Water Ext) of WB 

From each sample, 500 μL was transferred to a glass vial and a 1:1 MeOH:CHCl_3_ mixture (1 mL) was added. Samples were vortexed then a 1:1 MeOH:CHCl_3_mixture (500 μL) was added and samples were vortexed again. Ice-cold DI water (1 mL) was added in two aliquots (500 μL), samples were vortexed after each addition. Samples were centrifuged (1300× *g*, 4 °C for 20 min), and the aqueous fraction of each sample was transferred to a glass 2-dram vial for lyophilization. After lyophilization samples were resuspended in D_2_O for NMR analysis. 

### 4.6. Methanol Chloroform Precipitation (MeOH:CHCl_3_ppt)

This protocol was modified from the MeOH:CHCl_3_ precipitation described by Gowda and Raftery [22]. Samples were thawed and 500 μL was transferred to a glass vial and a 1:1 MeOH:CHCl_3_ mixture (1 mL) was added for a 1:1:1 sample:MeOH:CHCl_3_ratio. Samples were sonicated for 2 min at 4 °C, incubated at −20 °C for 20 min, then centrifuged (13,400× *g*, 4 °C, for 30 min). Aqueous supernatant was transferred to a labeled microcentrifuge tube and dried by lyophilization. After lyophilization samples were resuspended in 600 μL of 50 mM sodium phosphate buffer in D_2_O for NMR analysis [17]. 

### 4.7. 1d-^1^H NMR

Sample volume was measured and recorded. For serum samples, calcium formate (50 μL) of known concentration was used as the internal standard; for WB, the internal standard was DSS-d_6_ (50 μL) of known concentration with 0.2% sodium azide. Sample pH was measured and corrected to between 6.5–7.5 by dropwise addition of 0.1 mM deuterium chloride or sodium deuteroxide. Samples were transferred to 5 mm 500 MHz precision NMR tubes (Wilmad Lab Glass, Vineland NJ) for assay. 

NMR spectra were acquired at the University of Michigan’s Biochemical NMR Core Laboratory on a Varian (now Agilent, Inc., Santa Clara, CA) 11.74 Tesla (500 MHz) NMR spectrometer with a VNMRS console operated by host software VNMRJ 4.0, and equipped with a 5-mm Agilent “One-probe.” NMR spectra were recorded using 32 scans of the first increment of a 1 H,1 H-NOESY (commonly referred to as a 1d-NOESY or METNOESY) pulse sequence [32]. Spectra were acquired at a room temperature of 295.45 ± 0.3 K. The NMR pulse sequence was as follows: A 1 s recovery delay, which includes a 990 ms saturation pulse of 80 Hz (gB1) induced field strength empirically centered on the water resonance, 2 calibrated 90° pulses, a mixing time (tmix) of 100 ms, a final 90° pulse, and an acquisition period of 4 s. Optimal excitation pulse widths were obtained by utilizing an array of pulse lengths as previously described [10]. 

NMR spectra of serum and WB were analyzed with Chenomx NMR Suite 8.2 (Edmonton, AB, Canada) software. The Processor module was used to phase shift, baseline correct and excise water from each spectrum as previously described [10]. Compounds were then identified and quantified using the profiler module of the software, which allows metabolites to be quantified relative to an internal standard of known concentration [10]. Data was scaled to correct for differences in initial sample volume before analysis. 

### 4.8. Test of Long-Term Storage on Human Serum

To test the impact of long-term storage (−80 °C) on human serum, six technical replicates of samples that underwent MeOH:CHCl_3_:water extractions after four to eight months of storage at −80 °C were extracted after storage at −80 °C (total storage times ranged from two years and ten months to three years and two months). 

### 4.9. Data/Statistical Analysis

Exploratory analysis of profiled sample conditions was examined by principal component analysis (PCA) using SIMCA (13.0.3). Metabolite concentration data were normalized using Metaboanalyst and normalized datasets were exported for further statistical analysis using GraphPad Prism 7.0 [33]. Pooled human serum was normalized by log transformation and auto-scaling, serum from sepsis patients was cube transformed and auto-scaled, human serum from storage experiments was cube transformed and range scaled; pig WB was log transformed and auto-scaled, and baboon WB was cube transformed and mean scaled, pig WB was log transformed and auto-scaled. Comparison of normalized metabolite concentration data between two conditions from sepsis patients, healthy serum from storage experiments were compared by a Student’s *t*-test. Standard deviations were not assumed to be the same between metabolites, multiple comparisons were corrected for using the Holm-Šídák method. For the comparison of three conditions, normalized metabolite concentration data were compared using ANOVA with Tukey’s multiple comparisons test, if applicable. Mean coefficient of variance (CoV) was calculated by dividing the mean of concentrations by the standard deviation for each metabolite in a given condition, then averaging CoVs across every commonly quantified metabolite. The average total metabolite recovery (TMR) was calculated by taking the sum of all metabolites commonly detected for each sample in a given condition, then averaging the sums to give a single TMR for a condition. Confidence intervals (95%) were calculated for CoV and TMR. Differences between TMR and CoV were compared by Student’s *t*-tests, or by ANOVA with Tukey’s post-hoc test, as applicable. 

## 5. Conclusions

A comprehensive series of experiments were performed on pooled samples from a variety of sources. MeOH precipitations with ultrafiltrations are a good strategy to use with serum samples. We confirmed that, at low temperatures (−80 °C), the serum metabolome after extraction does not change after long-term storage (3–4 years).

WB is a promising biofluid for metabolomics, the nature of its collection and the inclusion of the red blood cell metabolome make it highly useful for metabolomics experiments. MeOH:CHCl_3_ precipitations are a rapid, reproducible, and low-variance strategy to use with WB. 

As metabolomics as a field moves towards more quantification, accuracy in 1d-^1^H NMR metabolomics will rely on careful selection of macromolecule removal strategies and consideration of pre-analytical sample handling and storage. 

## Supplementary Materials

The following are available online at http://www.mdpi.com/2218-1989/8/4/93/s1, Table S1: Summary of data from pooled healthy human serum, Figure S1: Detailed annotated spectra of representative MeOH ppt + UF pooled healthy human serum, Figure S2: Representative spectra of ultrafiltered pooled healthy human serum, Figure S3: PCA plot of pooled healthy human serum, Table S2: Summary of concentration data from pooled human serum from sepsis patients, Figure S4: Representative 1d-^1^H NMR spectra of pooled human serum from sepsis patients, Figure S5: PCA plot of pooled human serum from sepsis patients, Table S3: Summary of concentration data from matched healthy human serum extracted after short- or long-term storage, Table S4: Summary of concentration data from pooled healthy pig WB, Figure S6: Representative 1d-^1^H NMR spectra of pooled healthy pig WB, Figure S7: PCA plot of pooled healthy pig WB, Table S5: Summary of concentration data from pooled healthy baboon WB, Figure S8: Representative 1d-^1^H NMR spectra of pooled healthy baboon WB, Figure S9: PCA plot of pooled healthy baboon WB, Methods S4.4 Methanol:chloroform:water extractions–detailed procedure.
