# Supplementary material for: Rapid, Reproducible, Quantifiable NMR Metabolomics: Methanol and Methanol: Chloroform Precipitation for Removal of Macromolecules in Serum and Whole Blood
Source: Metabolites. 2018 Dec 14;8(4):93. doi: 10.3390/metabo8040093 (PMC6316042; doi:10.3390/metabo8040093)
Supplement: Supplementary file 1 [file metabolites-08-00093-s001.pdf]

Supplemental Information

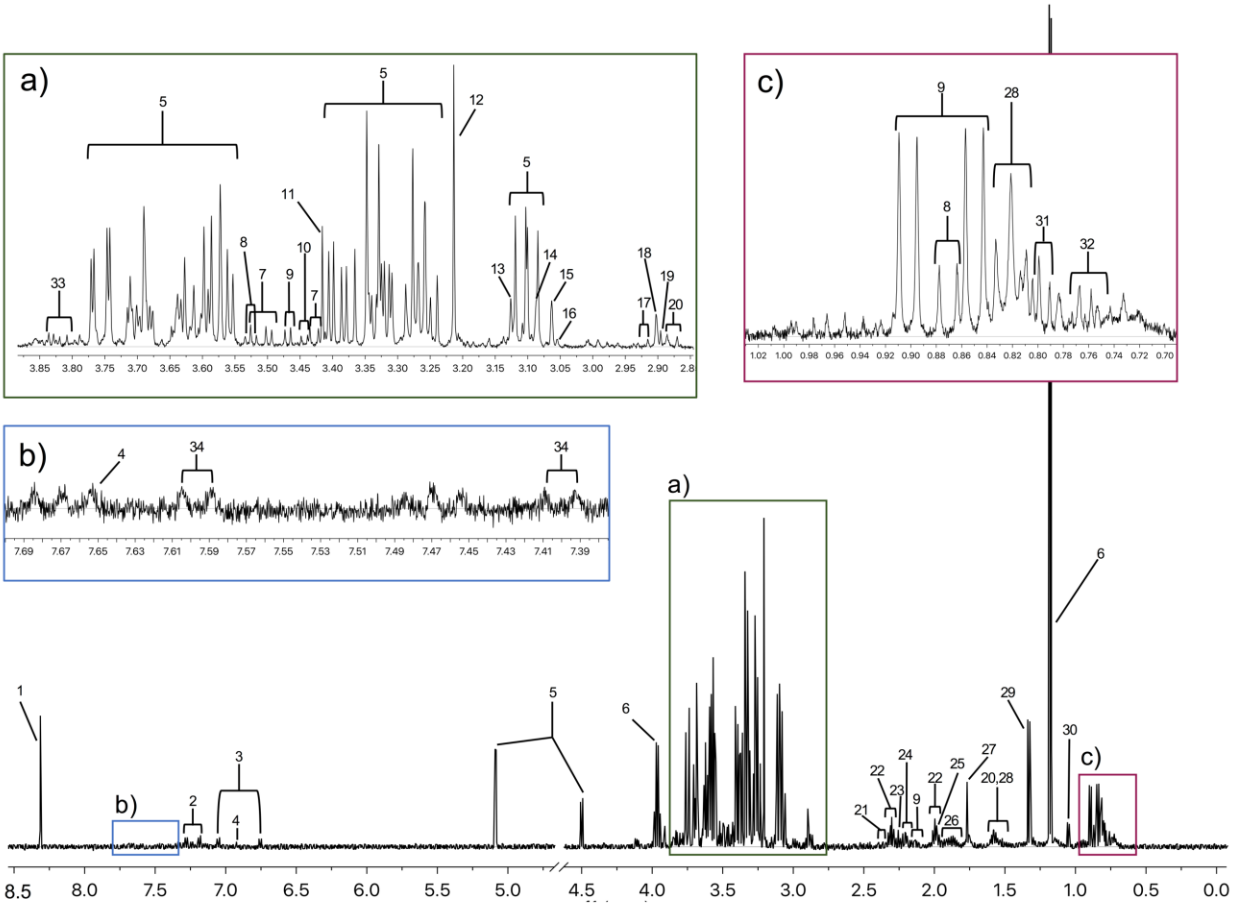

Figure S1. Representative annotated spectrum of a pooled normal human serum sample after MeOH ppt + UF. Compounds identified are: 1) formate (internal standard), 2) phenylalanine 3) tyrosine, 4) histidine, 5) glucose, 6) lactate, 7) glycerol, 8) isoleucine, 9) valine, 10) threonine, 11) glycine, 12) methanol, 13) betaine, 14) carnitine, 15) choline, 16) o-acetylcarnitine, 17) ornithine, 18) creatinine, 19) creatine, 20) lysine, 21) citrate, 22) glutamine, 23) succinate, 24) glutamate, 25) methionine, 26) proline, 27) acetate, 28) leucine, 29) alanine, 30) 3-hydroxybutyrate, 31) 2-oxoisocaproate, 32) 2-hydroxybutyrate, 33) serine, 34) tryptophan. KEGG IDs for quantified compounds are provided in Table S1.

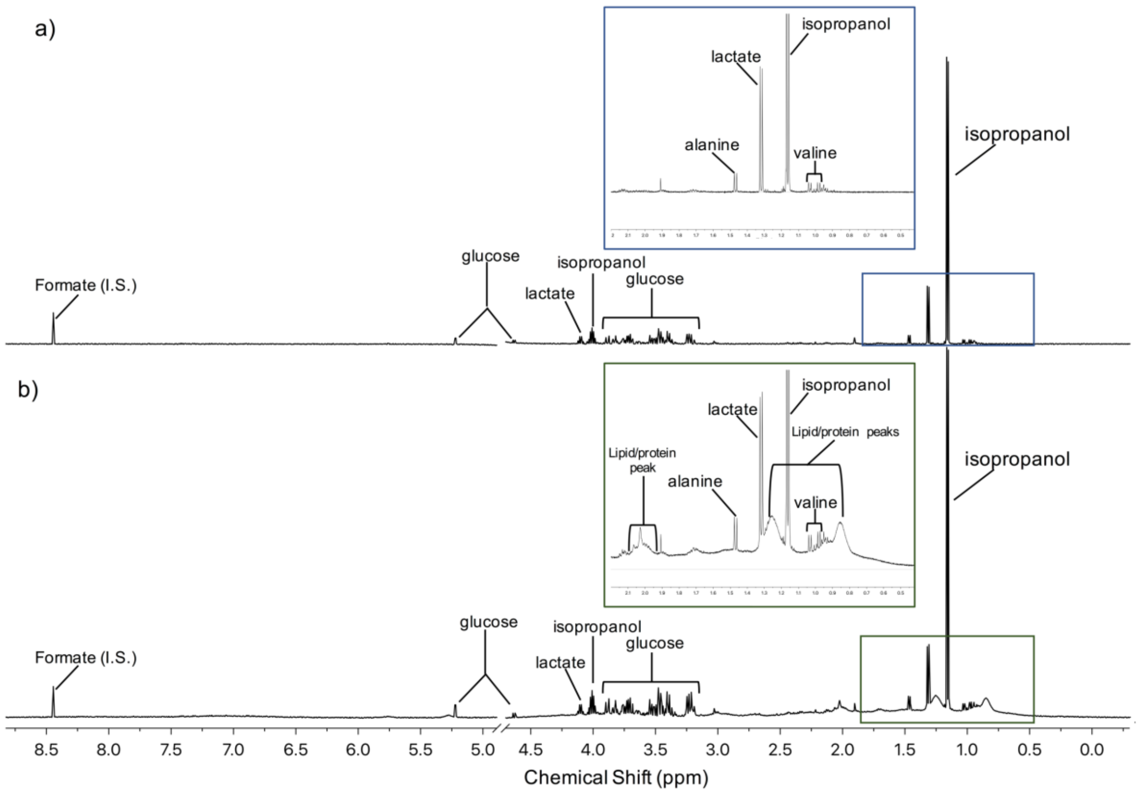

Figure S2. The variability in macromolecule removal is evident from representative 1D ^{1}H-NMR spectra of technical replicates of pooled normal human serum subjected to ultrafiltration only. (**a**) Is an example of a clean spectrum that can easily and reliably be quantified, while (**b**) shows visible macromolecule peaks across the spectrum, and particularly in the lower regions. Formate was added to samples as the internal standard (I.S.) and ^{1}H-NMR spectra were acquired on a 500MHz Varian NMR with metnoesy pulse sequence and 32 transients (see text for pulse sequence details).

Table S1. Summary of detected and quantified metabolites in healthy pooled human serum subjected to MeOH ppt + UF (n=20), MeOH:$CHCl_3$:water ext + UF (n=10), and MeOH ppt only (n=8). P-values were determined by ANOVA with Tukey's post-hoc test (adjusted p-value), performed on normalized data corrected for multiple comparisons.

		MeOH ppt + UF		MeOH:CHCl3:water ext + UF		MeOH ppt only		MeOH ppt+UF vs. MeOH ppt only	MeOH ppt+UF vs. MeOH:CHCl3:water Ext+UF	MeOH ppt only vs. MeOH:CHCl3:water Ext+UF
**Metabolites**	**KEGG ID**	**Median (uM)**	**IQR**	**Median (uM)**	**IQR**	**Median (uM)**	**IQR**	**Adjusted p-value**	**Adjusted p-value**	**Adjusted p-value**
2-Hydroxybutyrate	C05984	24.07	3.06	15.53	2.75	22.34	4.08	0.78	<0.0001	0.00
2-Oxoisocaproate	C00233	16.64	4.72	ND		11.73	2.05	0.28		0.0002*
3-Hydroxybutyrate	C01089	62.72	6.41	36.75	11.16	55.04	8.13	0.19	<0.0001	0.00
Alanine	C00041	364.33	28.90	331.48	20.14	332.58	58.09	0.00	0.02	0.84
Betaine	C00719	31.75	2.76	27.90	2.31	30.13	7.14	0.04	0.00	0.90
Carnitine	C00318	25.23	1.88	18.25	1.29	27.14	12.17	0.91	<0.0001	0.00
Choline	C00114	39.21	2.69	34.61	1.97	38.14	5.32	0.04	0.02	1.00
Citrate	C00158	26.93	11.39	73.29	25.55	29.47	8.10	0.71	<0.0001	0.00
Creatine	C00300	21.97	2.32	17.31	3.96	19.96	4.68	0.02	<0.0001	0.17
Creatinine	C00791	60.39	4.32	39.82	7.51	55.00	8.63	0.06	<0.0001	0.01
Glucose	C00221	3603.06	307.19	3176.94	251.09	3302.11	580.65	0.00	0.02	0.62
Glutamate	C00025	170.31	15.98	110.59	22.86	164.07	28.50	0.21	<0.0001	0.00
Glutamine	C00064	336.97	45.42	272.13	47.44	308.31	62.22	0.01	<0.0001	0.67
Glycerol	C00116	134.89	11.12	134.91	19.29	127.70	20.66	0.06	0.16	0.88
Glycine	C00037	277.38	35.02	266.70	28.27	246.10	49.93	0.00	0.27	0.11
Histidine	C00135	37.35	14.02	26.63	9.23	21.59	7.98	<0.0001	0.01	0.20
Isoleucine	C00407	62.84	3.81	50.44	8.52	57.57	8.43	0.06	<0.0001	0.11
Lactate	C00186	1999.61	145.15	1465.28	305.29	1832.62	323.62	0.03	<0.0001	0.09
Leucine	C00123	114.72	10.14	86.09	18.70	105.90	18.18	0.08	<0.0001	0.05

Lysine	C00047	112.46	13.13	94.74	14.33	111.04	23.07	0.08	<0.0001	0.27
Methionine	C00073	22.00	4.36	20.01	4.59	19.83	3.40	0.06	0.12	0.91
O-Acetylcarnitine	C02571	4.93	0.92	4.82	1.88	5.09	0.90	0.80	0.96	0.70
Ornithine	C00077	46.29	6.69	41.02	6.86	45.28	10.83	0.34	0.06	0.81
Phenylalanine	C00079	88.82	8.16	56.96	8.46	78.77	19.31	0.07	<0.0001	0.01
Proline	C00148	175.86	15.02	129.15	24.47	156.93	55.62	0.00	<0.0001	0.84
Serine	C00065	133.91	16.79	105.10	29.10	114.31	37.39	0.02	0.00	0.79
Succinate	C00042	11.18	1.80	8.77	0.96	10.04	2.95	0.22	0.25	0.98
Threonine	C00188	90.88	11.66	89.09	9.36	99.14	15.11	0.99	0.06	0.18
Tryptophan	C00078	45.69	5.83	ND		42.86	4.94	0.57		0.06*
Tyrosine	C00082	51.34	7.96	42.66	7.60	45.69	7.71	0.05	0.00	0.84
Valine	C00183	175.92	10.93	149.64	23.46	165.90	21.80	0.02	<0.0001	0.53

* t-test using Holm-Sidak correction for multiple comparisons performed on normalized concentrations of MeOH ppt + UF and MeOH ppt samples.

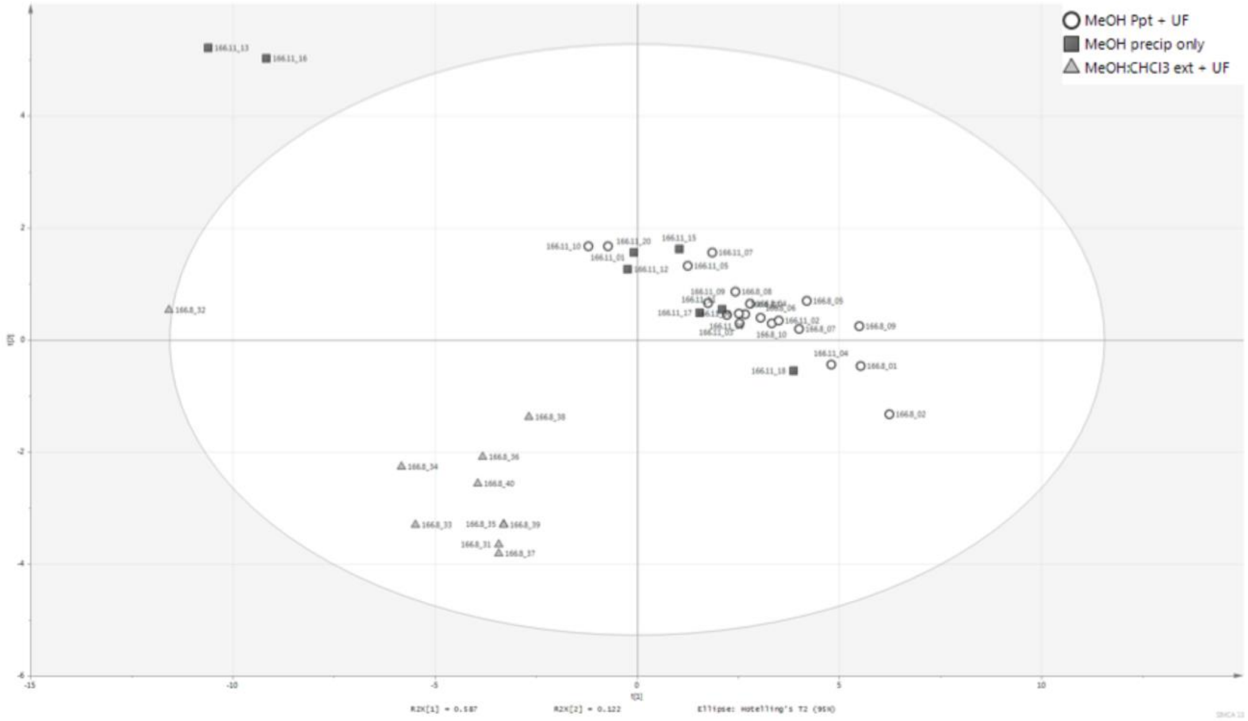

Figure S3. Principal component analysis (PCA) plots of technical replicate samples of pooled healthy human serum subjected to MeOH ppt + UF (n=20), MeOH:$CHCl_3$:water ext + UF (n=10), and MeOH ppt only (n=8); which shows that MeOH ppt + UF and MeOH are similar, while MeOH:$CHCl_3$:water ext + UF samples are distinct. MeOH ppt + UF samples show less variance than MeOH ppt samples or MeOH:$CHCl_3$:water ext + UF samples.

Table S2. Median metabolite concentrations (uM), and interquartile ranges (IQR) of metabolites detected and quantified in technical replicates of pooled serum from sepsis patients (n=4 for MeOH ppt + UF, n=5 for MeOH:$CHCl_3$:water ext + UF pooled human serum from sepsis patients). P-values determined by an unpaired Student's t-test, performed on normalized data, corrected for multiple comparisons using Holm-Sidak method (adjusted p-value).

		Methanol Precipitated +UF		Methanol:chlorform:water extracted+UF		
**Metabolite**	**KEGG ID**	**Median (μM)**	**IQR**	**Median (μM)**	**IQR**	**adjusted p-value**
2-hydroxybutyrate	C05984	77.15	5.23	74.64	7.63	1.00
3-Hydroxybutyrate	C01089	313.92	10.58	327.50	24.50	1.00
Alanine	C00041	200.72	9.94	208.07	18.95	1.00
Betaine	C00719	42.20	2.87	43.27	3.48	1.00
Carnitine	C00318	28.89	4.10	29.10	3.95	1.00
Choline	C00114	8.86	0.42	9.14	0.99	1.00
Citrate	C00158	22.34	0.14	53.10	3.84	0.09
Creatine	C00300	68.33	5.78	74.60	9.91	1.00
Creatinine	C00791	101.82	4.81	100.84	8.89	1.00
Glucose	C00221	4900.77	292.41	4996.33	200.17	1.00
Glutamate	C00025	57.92	10.65	51.77	11.26	1.00
Glutamine	C00064	295.55	11.33	311.90	23.54	0.99
Glycine	C00037	118.69	8.37	128.06	9.02	0.83
Histidine	C00135	22.10	5.19	24.35	3.17	1.00
Isoleucine	C00407	37.97	3.84	34.95	2.81	0.98
Lactate	C00186	1660.08	41.33	1812.24	60.94	0.80
Leucine	C00123	66.40	6.33	73.62	6.34	1.00
Lysine	C00047	78.99	3.25	78.44	10.19	1.00
Methionine	C00073	19.05	3.42	17.32	0.60	0.95
O-Acetylcarnitine	C02571	10.64	0.81	11.67	3.17	1.00
Ornithine	C00077	38.51	1.17	38.89	7.44	1.00
Phenylalanine	C00079	68.88	5.03	63.12	10.51	1.00
Proline	C00148	109.71	17.86	109.78	14.02	1.00
Pyruvate	C00022	66.93	5.21	37.10	7.72	0.01
Threonine	C00188	51.63	4.16	48.49	14.82	1.00
Tyrosine	C00082	45.82	2.95	48.75	3.36	1.00
Valine	C00183	116.33	8.89	121.22	11.24	1.00

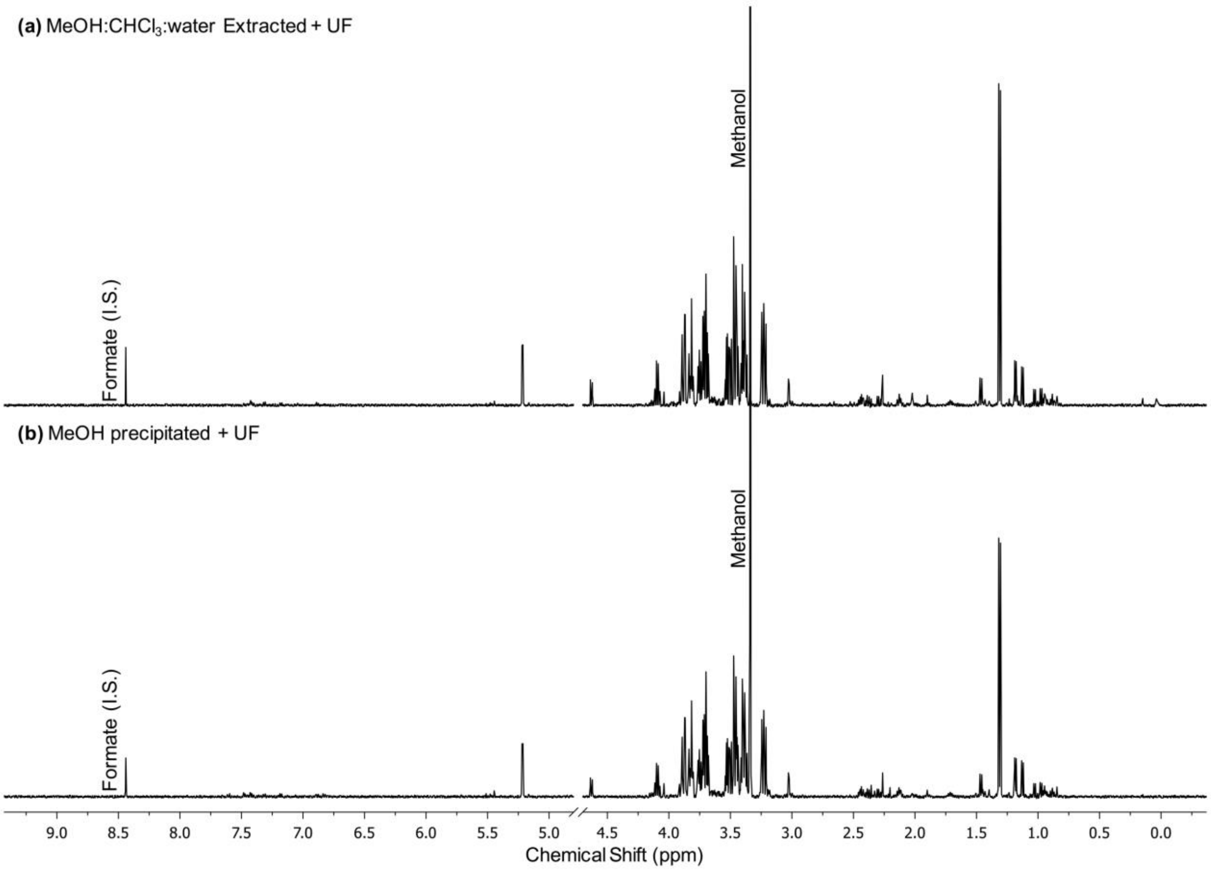

Figure S4. Representative 1D ^{1}H-NMR spectra of technical replicates of pooled human serum from patients with sepsis subjected to either (**a**) MeOH:$CHCl_3$:water extraction followed by ultrafiltration (+UF) or (**b**) MeOH precipitation + UF. Formate was added to samples as the internal standard (I.S.) and ^{1}H-NMR spectra were acquired on a 500MHz Varian NMR with metnoesy pulse sequence and 32 transients (see text for pulse sequence details). Phase shift correction, excision of the water peak, and baseline correction were performed before identification and quantification of metabolites (see text for details).

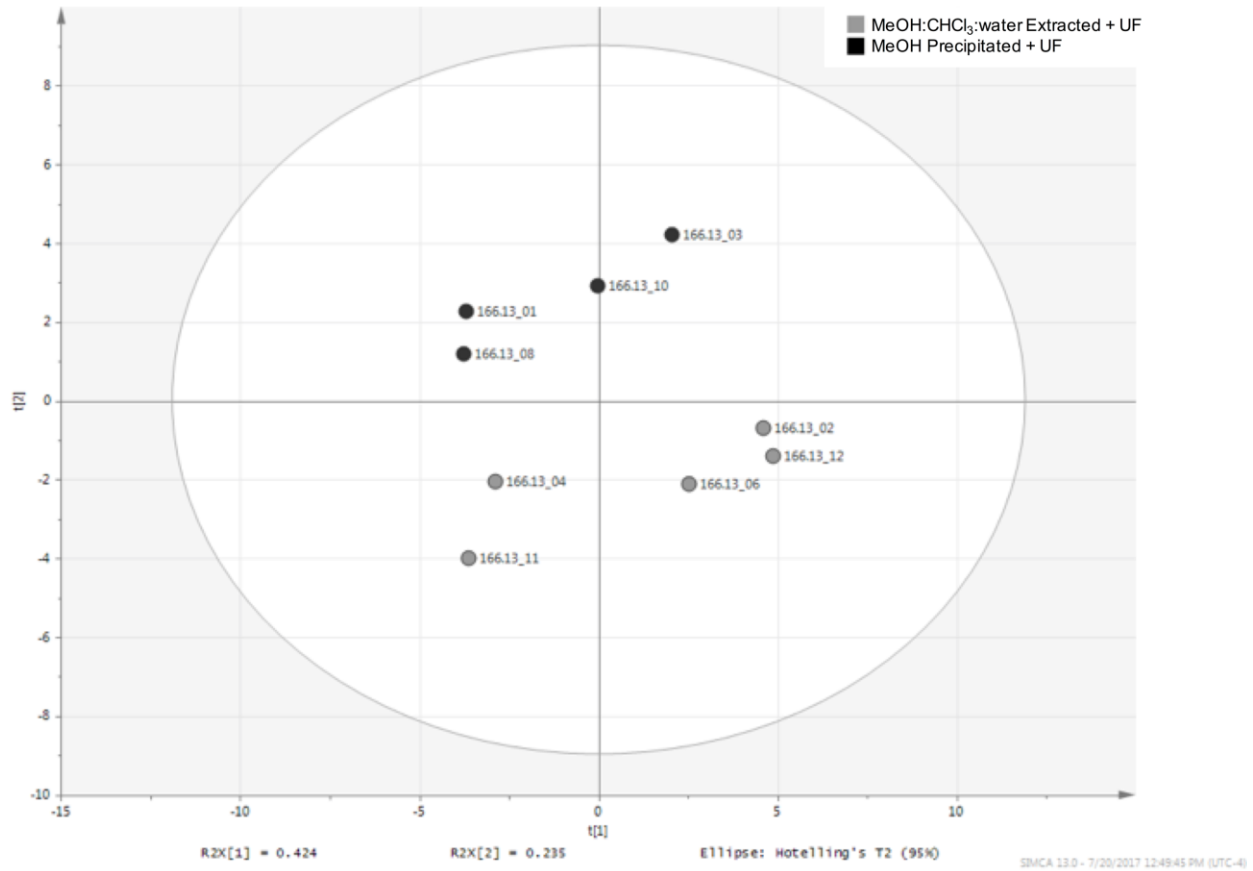

Figure S5. Principal component analysis (PCA) plots of technical replicate samples of serum from patients with sepsis subjected to MeOH ppt + UF (n=4) and MeOH:$CHCl_3$:water ext + UF (n=5), which shows that MeOH ppt + UF samples are distinct from MeOH:$CHCl_3$:water extraction + UF.

Table S3. Median metabolite concentrations (uM), and interquartile ranges (IQR) of metabolites detected and quantified in normal human serum after short term storage (<8 months) and long term (3-4 years) storage at -80°C (n=8 for both groups). P-values were determined by an unpaired Student's t-test, performed on normalized data corrected for multiple comparisons using Holm-Sidak method (adjusted p-value).

		Short term Storage at -80°C		Long term Storage at -80°C		
**Metabolite**	**KEGG ID**	**Median (µM)**	**IQR**	**Median (µM)**	**IQR**	**adjusted p-value**
3-Hydroxybutyrate	C01089	28.89	42.40	26.99	25.18	1.00
Alanine	C00041	186.50	56.26	173.11	56.12	1.00
Betaine	C00719	20.57	5.07	19.98	6.90	1.00
Carnitine	C00318	15.42	7.97	12.33	6.03	0.68
Choline	C00114	3.75	2.03	4.09	1.68	1.00
Citrate	C00158	55.76	14.50	50.79	8.96	1.00
Creatine	C00300	14.52	12.98	15.57	9.08	1.00
Creatinine	C00791	29.12	6.41	25.24	3.83	1.00
Glucose	C00221	2333.39	266.42	2319.15	93.48	1.00
Glutamate	C00025	34.54	18.24	46.63	12.99	1.00
Glutamine	C00064	298.40	33.47	286.18	24.70	1.00
Glycine	C00037	116.68	11.51	98.51	31.47	1.00
Histidine	C00135	19.10	12.71	30.34	2.89	0.49
Isoleucine	C00407	26.88	7.12	29.38	7.03	1.00
Lactate	C00186	758.11	341.37	654.79	419.17	0.97
Leucine	C00123	53.48	15.18	53.46	12.97	1.00
Lysine	C00047	74.43	14.62	70.86	12.13	1.00
Methionine	C00073	15.29	3.63	18.19	6.44	1.00
O-Acetylcarnitine	C02571	4.82	1.39	4.68	0.97	1.00
Ornithine	C00077	23.78	7.25	29.01	2.24	1.00
Phenylalanine	C00079	35.94	3.78	31.20	2.30	0.59
Proline	C00148	95.14	33.09	106.11	41.16	1.00
Propylene glycol	C00583	20.07	10.83	21.95	9.96	1.00
Serine	C00065	45.21	8.92	62.43	13.42	1.00
Threonine	C00188	47.30	8.83	53.47	11.38	1.00
Tryptophan	C00078	22.43	3.03	ND		
Tyrosine	C00082	32.46	4.93	32.94	5.83	1.00
Valine	C00183	102.69	38.53	91.66	27.48	1.00

Table S4. Median metabolite concentrations (uM) and interquartile ranges (IQR) of metabolites detected and quantified in technical replicates of pooled healthy pig whole blood (n=10 for MeOH:$CHCl_3$ precipitated samples, n=10 for MeOH:$CHCl_3$:water extracted samples). P-values were determined by an unpaired Student's t-test performed on normalized data corrected for multiple comparisons using Holm-Sidak method (adjusted p-value).

		**MeOH:$CHCl_3$ Precipitated**		**Methanol:chlorform:water extracted**		
**Metabolite**	**KEGG ID**	**Median (µM)**	**IQR**	**Median (µM)**	**IQR**	**adjusted p-value**
2-Oxoisocaproate	C00233	22.03	4.57	22.73	2.67	0.98
ADP	C00008	117.98	14.68	93.83	22.83	0.24
AMP	C00020	112.20	16.02	91.53	12.11	0.07
ATP	C00002	92.91	23.10	59.78	24.14	0.06
Alanine	C00041	312.17	37.50	281.62	29.87	0.27
Betaine	C00719	151.87	20.61	134.95	12.64	0.07
Choline	C00114	47.66	5.30	42.22	5.30	0.17
Creatine	C00300	181.09	28.75	168.26	17.45	0.62
Creatinine	C00791	64.89	6.50	65.06	4.86	0.98
Glucose	C00221	472.44	61.12	594.80	98.58	0.03
Glutamate	C00025	228.22	18.22	214.89	26.26	0.21
Glutamine	C00064	187.45	16.93	171.72	21.54	0.26
Glutathione	C00051	136.96	21.18	122.92	13.78	0.80
Glycine	C00037	645.39	65.16	606.51	69.02	0.70
Histidine	C00135	77.78	6.65	93.85	8.78	0.01
Hypoxanthine	C00262	138.42	9.20	187.39	42.72	0.00
IMP	C00130	94.95	14.47	89.90	12.06	0.87
Isoleucine	C00407	80.02	15.25	71.70	10.86	0.86
Lactate	C00186	5950.97	636.86	5221.06	502.94	0.10
Leucine	C00123	151.40	23.21	147.03	18.63	0.95
Lysine	C00047	269.23	43.59	225.97	8.67	0.03
Malonate	C00383	163.71	15.04	138.60	13.34	0.02
Methionine	C00073	33.10	5.29	30.55	3.26	0.95
Ornithine	C00077	126.23	17.68	112.11	4.52	0.05
Phenylalanine	C00079	59.19	3.76	62.93	6.33	0.74
Proline	C00148	260.61	53.53	249.50	30.76	0.80
Pyruvate	C00022	296.65	41.46	157.24	63.81	0.00
Serine	C00065	110.96	5.66	97.40	18.08	0.47
Succinate	C00042	16.52	1.73	15.14	2.87	0.95
Taurine	C00245	206.57	56.23	201.65	34.14	0.87
Threonine	C00188	129.63	20.48	123.16	18.64	0.98

Trimethylamine N-oxide	C01104	22.14	2.52	27.46	3.12	0.00
Tryptophan	C00078	33.16	5.24	38.97	9.69	0.55
Tyrosine	C00082	73.48	12.19	73.09	7.26	0.98
Valine	C00183	203.05	25.92	189.09	24.36	0.80

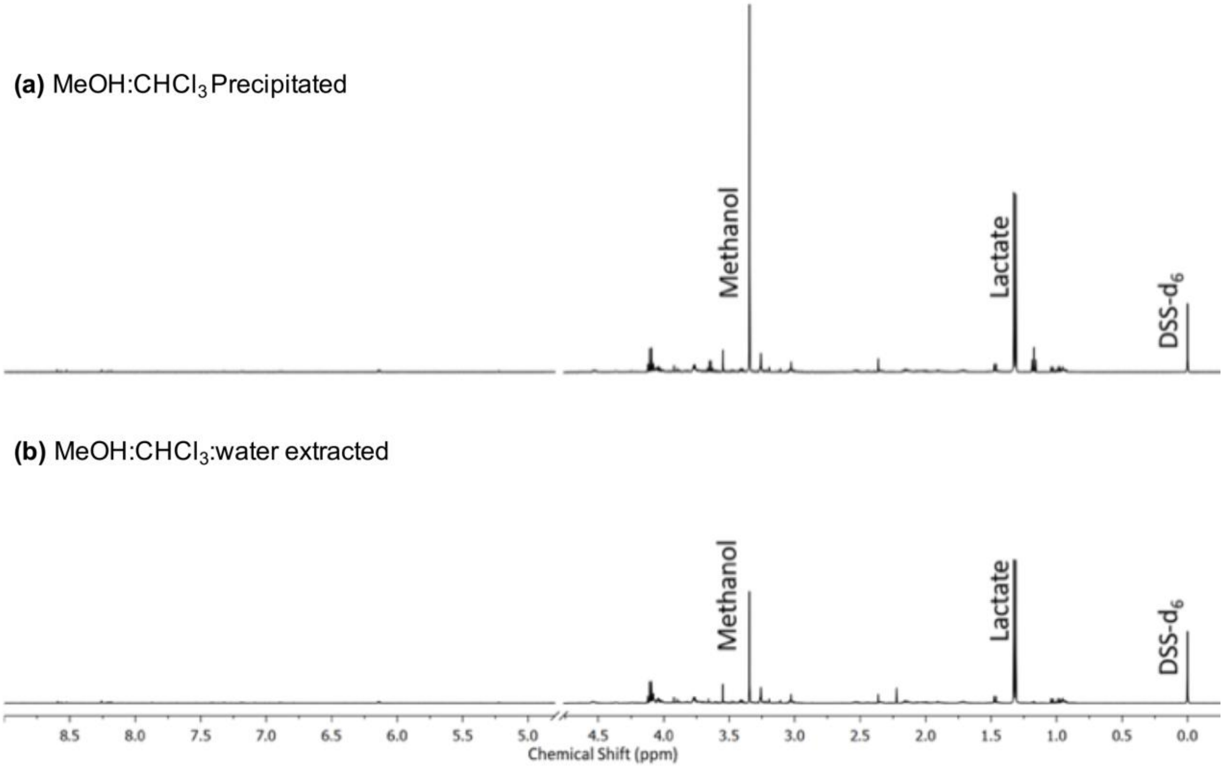

Figure S6. Representative 1D ^{1}H-NMR spectra of technical replicate samples of pooled healthy pig whole blood (WB) subjected to **a** MeOH:$CHCl_3$ precipitation, and **b** MeOH:$CHCl_3$:water extraction. ^{1}H-NMR were acquired on a 500MHz Varian NMR with metnoesy pulse sequence and 32 transients (see text for pulse sequence details). Phase shift correction, water peak excision, and baseline correction were performed before identification and quantification of metabolites (see text for details).

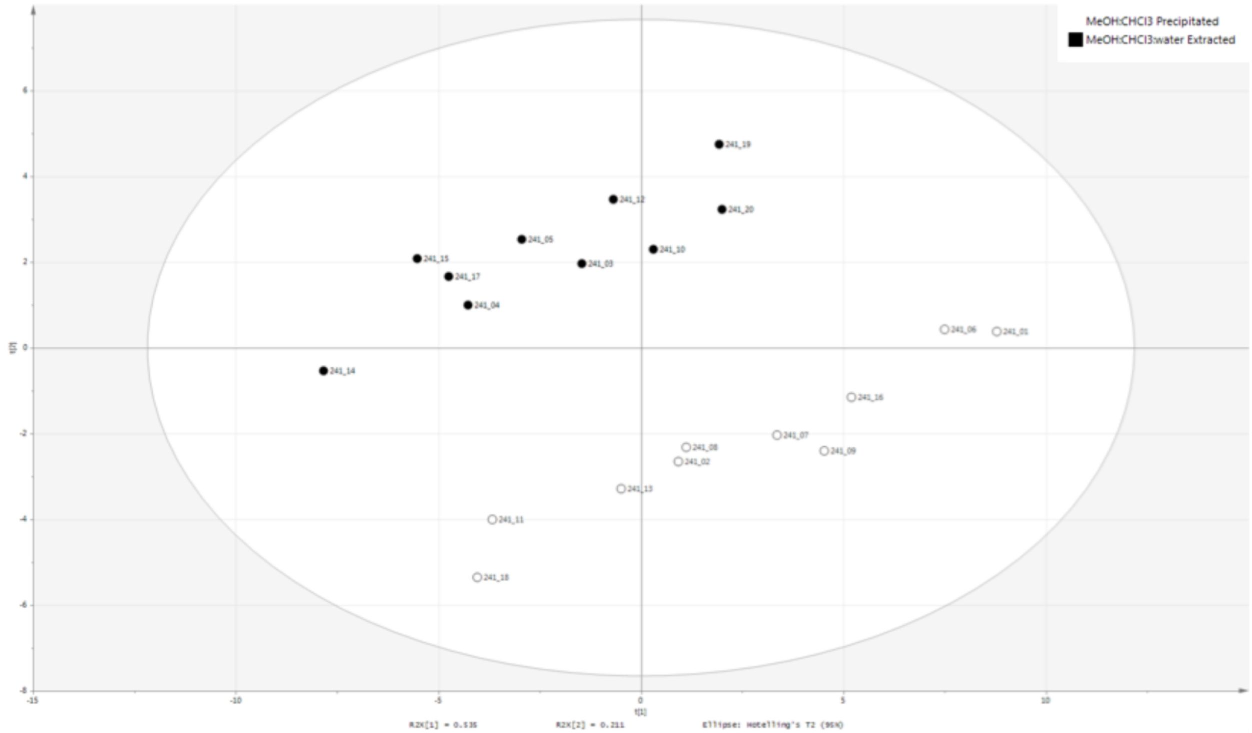

Figure S7. Principal component analysis (PCA) plots of technical replicates of pooled healthy pig whole blood (WB) which shows that MeOH:$CHCl_3$ precipitation (n=10) and MeOH:$CHCl_3$:water extraction (n=10) are distinct from each other, and have similar variance.

Table S5. Median metabolite concentrations (μM), interquartile ranges (IQRs), and results of two-way ANOVA comparing normalized concentration values in detected and quantified metabolites acquired from MeOH:$CHCl_3$ precipitated (n=4), MeOH:$CHCl_3$:water ext +UF (n=10), and abbreviated MeOH:$CHCl_3$:water ext (n=4) of pooled baboon whole blood (WB) samples. Multiple comparisons were done using Tukey's post-hoc test (adjusted p-value).

		**MeOH:$CHCl_3$ ppt**		**MeOH:$CHCl_3$:water ext + UF**		**Abbreviated MeOH:$CHCl_3$:water ext**		**MeOH:$CHCl_3$:water Ext+UF vs MeOH:$CHCl_3$ ppt**	**MeOH:$CHCl_3$:water Ext+UF vs Abbreviated MeOH:$CHCl_3$:water Ext**	**Abbreviated MeOH:$CHCl_3$:water Ext vs MeOH:$CHCl_3$ ppt**
**Metabolite**	**KEGG ID**	**Median (μM)**	**IQR**	**Median (μM)**	**IQR**	**Median (μM)**	**IQR**	**adjusted p-value**	**adjusted p-value**	**adjusted p-value**
2-Oxoisoaproate	C00233	10.82	1.22	17.50	3.53	10.82	3.22	0.35	0.46	1.00
3-hydroxybutyrate	C01089	41.96	6.71	68.82	21.18	49.11	6.07	0.01	0.03	0.98
3-Hydroxyisobutyrate	C01188	14.02	3.40	23.87	5.61	15.28	3.14	0.12	0.01	0.64
3-Methyl-2-oxovalerate	C03465	12.35	0.82	18.01	2.01	14.03	2.42	0.68	0.83	0.98
ADP	C00008	71.43	9.65	41.35	16.49	47.91	7.48	0.00	0.72	0.03
Alanine	C00041	123.96	8.14	198.42	24.27	137.56	25.86	<0.0001	0.00	1.00
AMP	C00020	153.40	2.16	170.50	24.93	128.38	38.91	0.73	0.02	0.16
Arginine	C00062	237.29	36.19	523.08	127.33	328.00	48.78	<0.0001	<0.0001	0.32
Aspartate	C00049	60.00	7.77	92.77	18.98	51.74	13.79	0.00	0.00	0.80
ATP	C00002	70.10	3.71	117.67	30.30	58.01	38.99	0.00	<0.0001	0.33
Betaine	C00719	47.71	1.55	73.92	8.25	51.56	9.92	0.01	0.05	0.95
Choline	C00114	10.72	0.42	17.40	5.10	10.27	3.52	0.14	0.15	0.99
Creatine	C00300	43.92	0.77	70.40	13.45	46.43	11.06	0.02	0.03	1.00
Creatinine	C00791	21.24	0.19	49.65	6.02	32.18	8.95	<0.0001	0.02	0.45
Glucose	C00221	2227.63	94.64	4090.37	609.89	2304.12	606.69	<0.0001	<0.0001	0.79
Glutamate	C00025	246.33	13.05	424.13	56.19	256.04	62.21	<0.0001	<0.0001	0.95
Glutamine	C00064	231.24	19.48	360.43	55.19	220.62	59.21	<0.0001	<0.0001	0.82
Glutathione	C00051	184.27	14.87	312.99	30.36	243.80	32.90	<0.0001	0.01	0.28

Glycine	C00037	268.54	10.00	362.80	64.13	269.63	59.15	0.00	0.00	0.86
Histidine	C00135	36.80	3.80	65.52	13.30	45.95	12.80	0.00	0.02	0.90
IMP	C00130	36.91	2.41	49.80	12.67	37.44	17.05	0.18	0.11	0.95
Isoleucine	C00407	37.22	2.23	62.70	13.76	42.11	9.57	0.00	0.02	0.95
Lactate	C00186	2260.21	167.98	3744.56	467.26	2072.07	593.70	<0.0001	<0.0001	0.03
Leucine	C00123	63.92	4.89	100.06	15.47	57.36	24.37	0.00	0.00	0.82
Lysine	C00047	154.85	13.40	247.35	11.55	163.03	41.27	<0.0001	<0.0001	1.00
Malonate	C00383	112.06	6.80	188.35	13.93	136.64	26.44	<0.0001	0.00	0.70
O-Acetylcarnitine	C02571	11.96	0.72	22.39	2.56	14.13	2.03	0.04	0.08	0.99
Phenylalanine	C00079	31.44	7.26	61.01	14.67	35.53	14.21	0.00	0.01	0.93
Proline	C00148	95.67	0.77	108.31	17.25	81.22	23.35	0.90	0.00	0.03
Pyruvate	C00022	75.83	8.23	85.20	25.97	72.51	18.16	0.60	0.46	0.96
Serine	C00065	95.26	3.30	127.06	14.89	66.00	15.39	0.09	<0.0001	0.03
Taurine	C00245	147.84	17.85	271.64	59.03	161.43	26.31	<0.0001	<0.0001	0.86
Trimethylamine N-oxide	C01104	27.73	5.46	74.46	13.20	40.40	5.79	<0.0001	0.00	0.31
Tyrosine	C00082	31.46	3.51	57.45	14.61	38.38	7.96	0.00	0.05	0.82
Valine	C00183	91.03	8.06	167.95	46.54	98.55	25.40	<0.0001	<0.0001	0.97

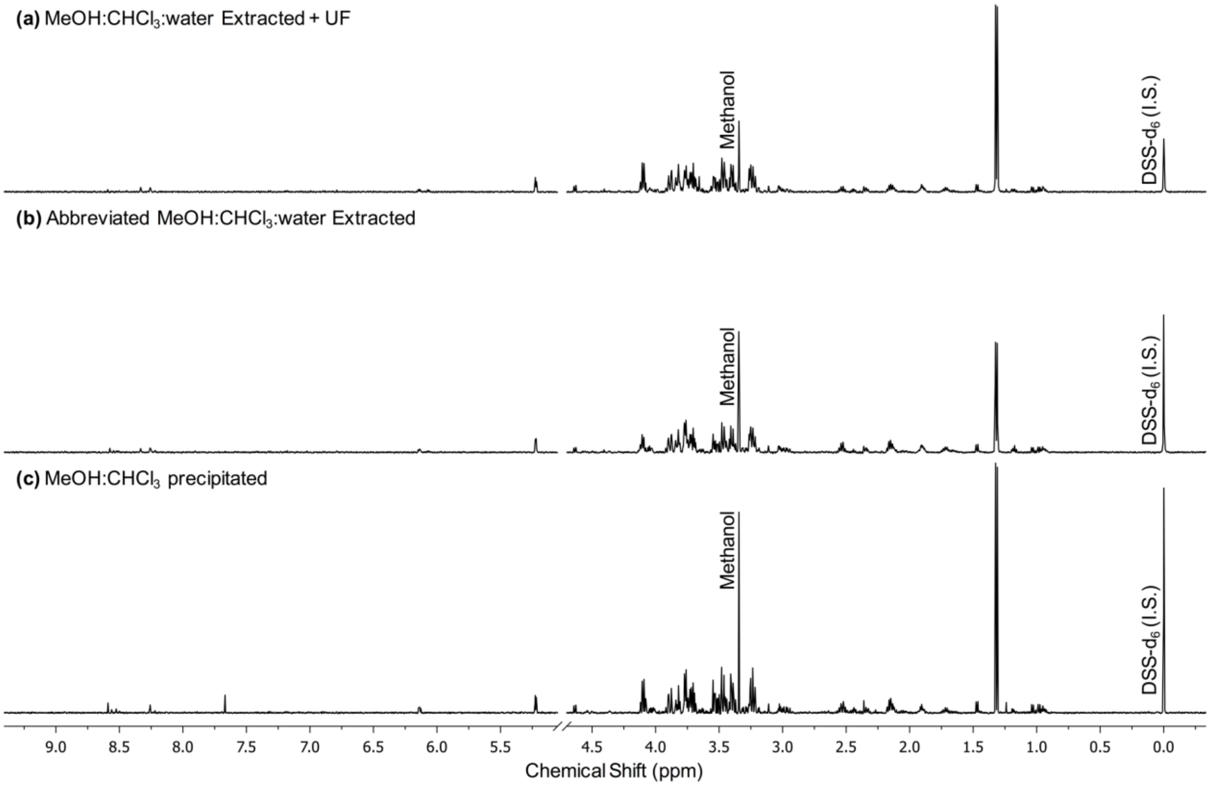

Figure S8. Representative 1D ^{1}H-NMR spectra of technical replicate samples of pooled baboon whole blood (WB) subjected to (**a**) MeOH:$CHCl_3$:water extraction followed by ultrafiltration (+ UF), (**b**) MeOH:$CHCl_3$ precipitation, or (**c**) abbreviated MeOH:$CHCl_3$:water extraction. 1D ^{1}H-NMR were acquired on a 500MHz Varian NMR with metnoesy pulse sequence and 32 transients. Phase shift correction, water peak excision, and baseline correction were performed before identification and quantification of metabolites (see text for details).

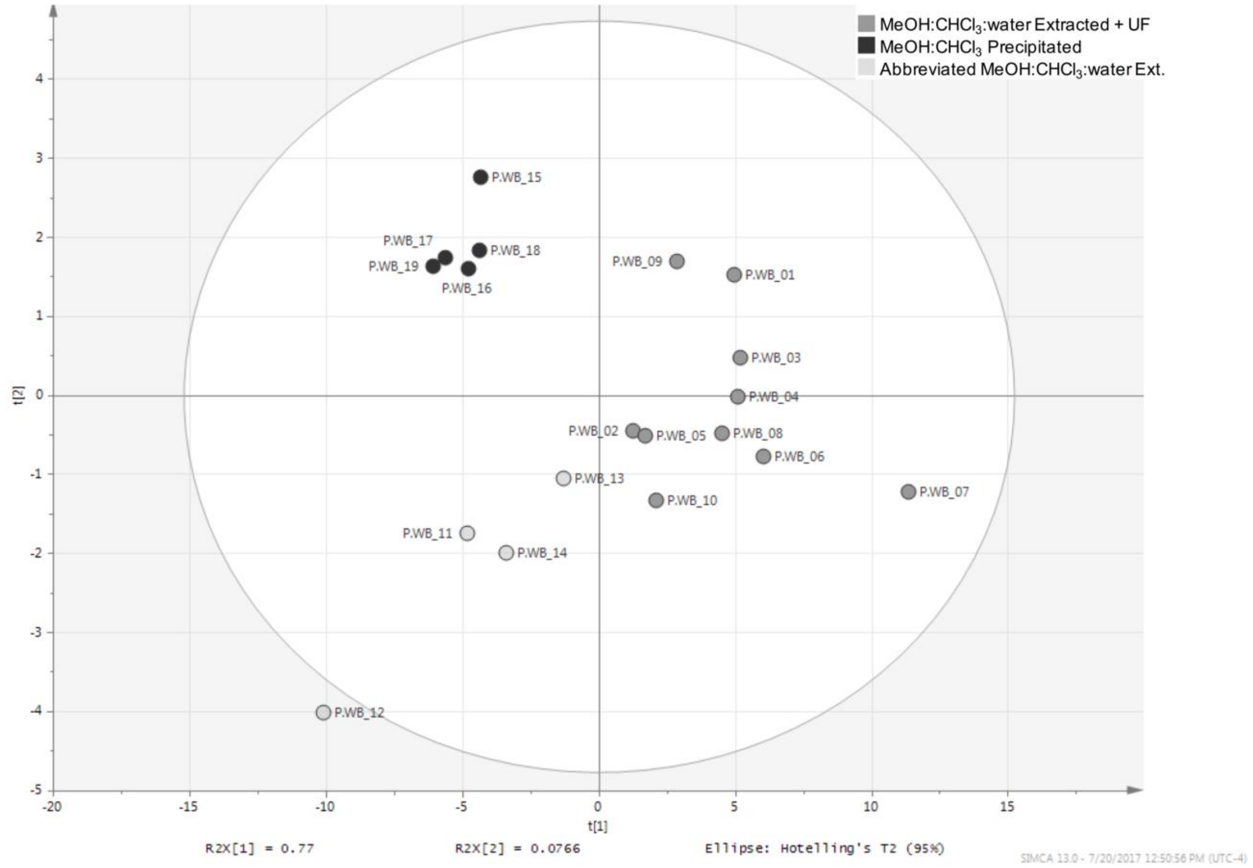

Figure S9. Principal component analysis (PCA) plots of technical replicates of pooled baboon whole blood (WB) which shows that variance is less when MeOH:$CHCl_3$ precipitation is used compared to MeOH:$CHCl_3$:water extracted followed by ultrafiltration (+UF) or abbreviated MeOH:$CHCl_3$:water extracted samples.

S4.4 Methanol:chloroform:water extractions – detailed procedure

From each sample, 500 µL was transferred to a glass vial and extracted with MeOH:$CHCl_3$ (2 mL, 1:1 v/v), vortexed, then centrifuged (1300*g*, 4°C for 20 min). Supernatants were transferred to a second vial, and the pellet was extracted with MeOH:$CHCl_3$ (1 mL), vortexed, and centrifuged (1300*g*, 4°C for 20 min). Supernatants from each extraction step were combined in a glass vial. Pellets were resuspended in 1mL deionized water. Supernatants were washed with 1mL water, vortexed, and chilled (4°C for 18 min). The upper (aqueous) layer was added to the pellet, vortexed, and centrifuged (1300*g*, 4°C for 20 min); the lower (organic) layer was dried by speed vacuum (50°C) for later analysis. The supernatant was removed from the pellet into a round-bottom flask (50-mL) and the pellet was washed with water (1 mL), vortexed, and centrifuged (1300*g*, 4°C for 20 min). This supernatant was added to round-bottom flask, and combined supernatants containing the aqueous metabolites were diluted with DI water (2 mL) and dried by lyophilization for no more than 24 hours.
